# On the Application of Information Geometry to the Manifold Induced by the Parameters of the Mean Square Error of Probability Functions

**DOI:** 10.3390/e28060667

**Published:** 2026-06-11

**Authors:** Argelia Aguilar Garduño, Candelaria Sansores Pérez, Antonio Neme

**Affiliations:** 1 Postgraduation Program in Computer Science, Universidad Nacional Autónoma de México, Mexico City 04510, Mexico; argelia29@comunidad.unam.mx; 2 Ciencias Básicas e Ingenierías, Universidad del Caribe, Cancún 77528, Mexico; csansores@ucaribe.edu.mx; 3 Instituto de Investigaciones en Matemáticas Aplicadas y en Sistemas (IIMAS), Universidad Nacional Autónoma de México (UNAM), Mexico City 04510, Mexico

**Keywords:** probability distribution, information geometry, mean square error, arc length

## Abstract

Some probability distributions can be described by relevant parameters, such as the mean and standard deviation for the case of Gaussians. This parameterization defines a manifold in which probability functions can be studied from a geometrical perspective. Information Geometry studies probability functions as points in this parameter-defined space, applying differential geometry. Probability functions can also be described by their mean square error, which can be approximated by a second-degree polynomial. In this contribution, we describe the characterization of probability functions in terms of the coefficients of second-degree polynomials that approximate their mean square error. The parameters of this polynomial define a manifold, approximated by a second-degree polynomial, in which probability distributions from different families can be compared by computing the arc length of the points linked to the distributions. One of the advantages of this approach is that the probability distributions can be compared in a more geometrical perspective. In this contribution, we describe the geometry of the induced manifold, and at the same time, we compare this manifold with the common structures from Information Geometry such as the Fisher–Rao distance. We offer empirical evidence that the characterization of probability distributions based on their mean square error can be of relevance not only for comparing them but also to gain a different look at the relation between probability distributions.

## 1. Introduction

A relevant task in statistics and machine learning is the comparison of probability distributions [1]. This problem has been tackled from a wide range of paths, including statistical tests and, closer to this contribution, an Information Geometry perspective [2,3]. In Information Geometry, a probability function is represented by a vector of parameters, and these parameters define a statistical manifold. In this manifold, the application of concepts from differential geometry helps unveil relevant properties of the distribution of points, representing probability distributions, such as geodesics [4], clusters [5], and anomalies [6].

**Figure 1:**
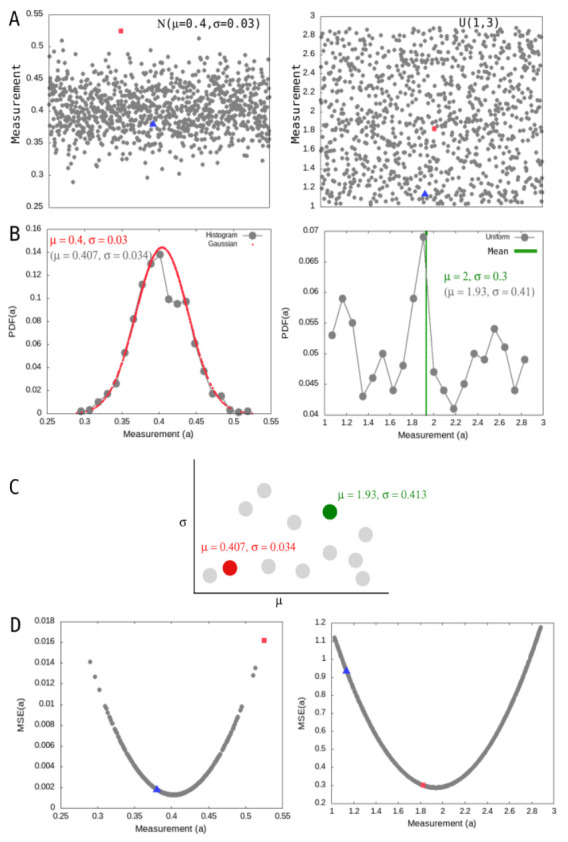
The Information Geometry approach to the comparison of distributions. (**A**) **Left**: A sample of 1000 observations withdrawn from a Gaussian distribution N(μ=0.4, σ=0.3), **right**: 1000 observations from a uniform distribution in the range [1,3]. Two measurements are indicated for both distributions. (**B**) The histogram computed from the samples is shown in gray. The empirical mean and standard deviations, computed from the samples, are shown in gray. (**C**) The mean (μ) and standard deviation (σ) for both distributions (green for the uniform one, red for Gaussian distribution) are plotted in the statistical manifold. In this manifold, the Fisher–Rao distance can be applied to compare distributions. (**D**) For each measurement a from the collection or random variable in A (x-axis), an error is presented (y-axis). This error is the mean square error, assuming that a is the actual true value.

Two random variables, representing a system in two different conditions or two processes compared at the same condition, can be compared from several perspectives. In Figure 1A left, a collection of 1000 samples or measurements was collected from a Gaussian distribution with parameters μ=0.4, σ=0.03. In the same figure, on the right, is a collection of 1000 samples withdrawn from a uniform distribution in the range (0,3). From each of these two collections, histograms can be computed, as shown in Figure 1B. There, the empirical parameters, obtained from the samples, are displayed: μ=0.407, σ=0.034 for the Gaussian distributions and μ=1.93, σ=0.41 for the uniform distributions. These two histograms can be compared in a number of ways, such as a Kolmogorov–Smirnoff test [7,8], by computing the 1-D Wasserstein distance or by calculating the Aitchison distance. In all three cases, the comparison is made directly over the histograms.

There are alternatives to compare histograms or probability functions. Some probability functions can be represented by their mean μ and standard deviation σ. These two parameters define a statistical manifold [2,4], and its geometry can be captured by the Fisher–Rao distance [9]. Probability distributions can be compared in this manifold using relevant distance functions or relying on relevant divergence metrics, as depicted in Figure 1C, where the parameters derived from the distributions are the coordinates in the manifold.

In this contribution, to compare probability distributions, we follow a different path to that of applying statistical tests, and closely related to Information Geometry, where one of the main objectives is that of computing distances in a statistical manifold. In Figure 1D, both left and right, the 1000 samples shown in Figure 1A are observed in the x-axis, and in the y-axis, an error associated with the measurement is shown. This error is the mean square error (MSE) associated with observation a, considering a as the best descriptor of the elements in the sample or collection. For clarity, two specific points, shown as a red square and a blue triangle, are shown for both distributions in Figure 1A,D. For the Gaussian distribution, it is observed that the red square has a large value (A), and thus the error associated with it is rather high, as shown in D. For the blue triangle, located near the mean of the distribution, the error is close to the minimum, which corresponds to the empirical mean. The opposite is observed for the uniform distribution, where the measurement for the red square is located near the mean, and thus its error is low, whereas the blue triangle presents a low measurement and thus a high error (D).

The core idea we tackled in this contribution is based on discrete probability distributions and can be described as follows. From a list L of observations $\left(x_{0},x_{1},\ldots ,x_{n-1}\right)$ depicting n measurements of a random variable, its probability distribution or its characterization as a histogram, P, is obtained. We characterize P via its mean square error considering each value a∈L as the true descriptor of the distribution, that is, the value with the minimum square error. We will refer to this characterization as MSE(P,a) or, if the context allows disregarding a, as MSE(P). MSE(P,a) refers to a function that assigns an error to each observation a∈L when that parameter a is considered the true value. MSE(P,a) presents some interesting properties. One such property is its characterization as a second-degree polynomial, and, in particular, the parameters of the polynomial are to be identified for the next steps in the analysis. We show that the linear and constant parameters of the polynomials of MSE(P,a) induce a manifold that can be represented by a second-degree polynomial. In this manifold, we can compare probability distributions from a different perspective and gain insight into the closeness or resemblance along the studied distributions.

**Figure 2:**
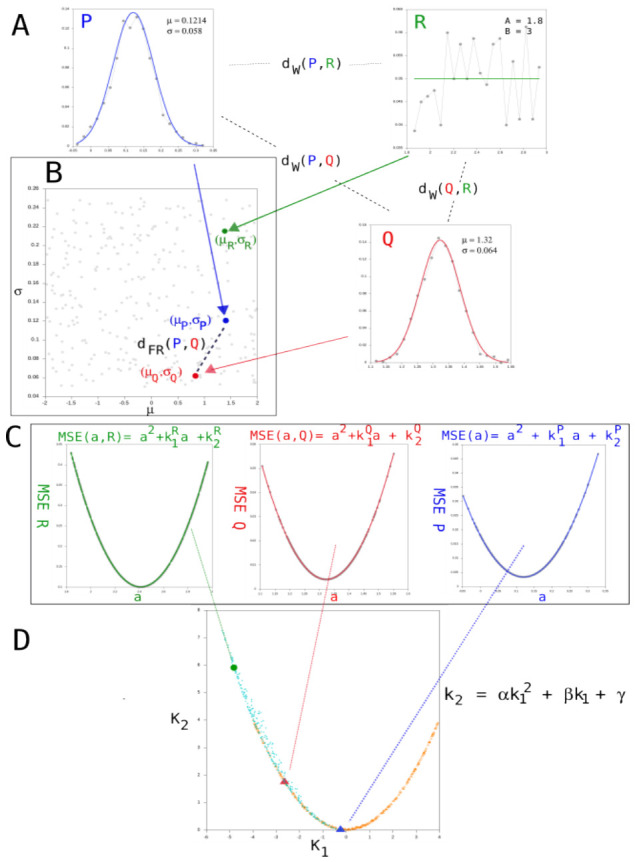
The overall idea of the comparison of probability functions by comparing the parameters of the polynomial that approximates their mean square error (MSE). Probability functions can be compared directly by the Wasserstein distance (**A**) or by computing the Fisher–Rao distance in the statistical manifold, induced by the mean and standard deviation, for Gaussian functions (**B**). The MSE of probability functions along range X is always a second-degree polynomial (**C**), and its linear and constant parameters define a manifold in which probability functions can be embedded and compared by computing the arc length along this polynomial (**D**).

Figure 2 shows the main argument of this contribution. In Figure 2A, three probability functions are depicted, namely P,Q,R. P comes from a Gaussian distribution with parameters N(μ=0.12, σ=0.5) depicted as a solid blue line, from which one hundred samples were withdrawn and shown as gray dots. Similarly, Q shows a Gaussian distribution with parameters N(μ=1.32, σ=0.06) displayed as a solid red line. Again, a hundred samples were withdrawn from that distribution and shown as gray dots. R is a uniform distribution in the range (1.8,3) or as N(μ=0.05, σ=0.01). The three histograms can be compared using the 1D Wasserstein distance ($d_{W}$) or the Aitchison distance [1,10]. The probability functions can be compared in the manifold defined by their parameters μ and σ (Figure 2B). In this statistical manifold, the Fisher–Rao distance ($d_{\mathrm{FR}}$) can be applied to compare the functions. However, we follow a different path. For a probability function P, its MSE(P) can be computed. In Figure 2C, for the distributions P,Q,R, their MSE(.) are shown as a function of the measurements a in each distribution. MSE can be represented by a second-degree polynomial, for which the quadratic parameter is always 1. As also shown in Figure 1D, the MSE of a distribution can be represented by a second-degree polynomial. In Figure 2C, the polynomial that fits the MSE of each of the three distributions P,Q,R is shown, with their corresponding equations: $\mathrm{MSE}\left(a,P\right)=a^{2}+k_{1}^{P}\times a+k_{1}^{P}$, $\mathrm{MSE}\left(a,Q\right)=a^{2}+k_{1}^{Q}\times a+k_{1}^{Q}$ and ($\mathrm{MSE}\left(a,R\right)=a^{2}+k_{1}^{R}\times a+k_{1}^{R}$). The parameters $k_{1}$ and $k_{2}$ are specific for each distribution but can be derived, as will be detailed in Section 2, from the mean and standard deviation of the distributions. The linear and constant parameters of the polynomial representing MSE define a manifold in which probability functions can be compared (Figure 2D). Here, each point comes from the parameters that define the second-degree polynomial of MSE. Orange dots represent Gaussian distributions, while green dots represent uniform distributions. This new manifold is also a second-degree polynomial. The comparison we suggest is conducted in the manifold defined by the linear (α) and constant β parameters of MSE.

In Figure 2D, each dot represents the MSE of a probability function. The green circle represents the uniform distribution, whereas the red and blue triangles represent the Gaussian distribution from Figure 2A. The rest of the points in Figure 2D are linked to Gaussian (orange triangles) or uniform (pale green circles) distributions.

The MSE takes into account in its definition the mean and the standard deviation. In that sense, our proposal is closely related to Information Geometry, where a statistical manifold, defined by precisely the mean and standard deviation of distributions, is the structure in which the analysis can be conducted.

The motivation of the approach presented in this contribution is that of comparing distributions or collections of limited data from a different perspective. The idea of transforming a distribution or a collection of observations to a representation based on the mean square error may be of interest, since it can capture, as we show here, interesting properties and can compare distributions under a different set of assumptions. In that sense, counting with such an approach can expand the set of available paths for comparing distributions.

This contribution continues as follows. In Section 2, the mean square error is described, focusing on the second-degree polynomial that defines it. In Section 3, we describe the relevant attributes of the manifold induced by the parameters of the second-degree polynomial of MSE. We continue to Section 4, where we offer computational evidence for the relevance of comparing probability functions in the manifold induced by the parameters of the polynomial fitting of the MSE. Finally, in Section 5, we offer some conclusions, limitations, and future work related to our contribution.

## 2. Mean Square Error and Its Approximation by Polynomials

The mean square error, or MSE, of a random variable X is defined as $\mathrm{MSE}\left(a\right)=E[{\left(X-a\right)}^{2}]$, where a is an estimator of the distribution [11,12]. Here, a is any value in the range R of X, and the MSE achieves the global minima at the mean of X [13]. In this sense, X can be represented by its MSE along values a∈R.

Let P be the discrete probability distribution obtained from a collection of observations L. Let a ∈ *L* be an observation or measurement, and let l=|L| be the cardinality of *L*. The MSE associated to each measurement a is defined as

(1) $$\mathrm{MSE}\left(P,a\right)=1/\left(l-1\right)\sum_{b\in L,b\neq a}{\left(a-b\right)}^{2}$$

The range of MSE is the same as the range of the probability distribution; that is, both have the same support. Although the minimum of MSE corresponds to the mean of L, we do not intend to replace L by a single scalar, for example, its mean. The rationale of computing the MSE for each value a∈L is to use it to represent L and its probability distribution in a different perspective and compare that representation with similar representations from other collections of observations.

The evaluation of MSE(a) in its support can be described by a second-degree polynomial. It is important to identify the coefficients of such polynomial in order to generate a new space, defined by those parameters, in which random variables can be compared following the proposal described in this contribution. The assertion that MSE(a) can be fitted to a second-degree polynomial constitutes the first lemma in the current contribution and we will proceed to offer a proof of it.

**Lemma** **1.** *The MSE(P,a) of a probability distribution P is represented by a second-degree polynomial with a quadratic term equal to 1*.

**Proof.**  To prove Lemma 1, we have to find a representation of MSE as a second-degree polynomial. A second-degree polynomial p evaluated in x∈X is of the form $p=k_{0}\times x^{2}+k_{1}\times x+k_{2}$. The representation of the second-degree polynomial for MSE is as follows:

(2) $$\begin{array}{c} \mathrm{MSE}\left(a\right)=E[{\left(X-a\right)}^{2}],\;\mathrm{from}\;\mathrm{the}\;\mathrm{definition}\;\mathrm{of}\;\mathrm{MSE}\\ \mathrm{MSE}\left(a\right)=E\left[X^{2}-2\mathrm{aX}+a^{2}\right],\;\mathrm{by}\;\mathrm{expanding}\;\mathrm{the}\;\mathrm{binomial}\\ =E\left[X^{2}\right]-2aE\left[X\right]+a^{2},\;\mathrm{by}\;\mathrm{the}\;\mathrm{linearity}\;\mathrm{of}\;\mathrm{the}\;\mathrm{expectation}\\ \mathrm{MSE}\left(a\right)=a^{2}-2aE\left[X\right]+E\left[X^{2}\right],\;\mathrm{after}\;\mathrm{rearranging}\;\mathrm{the}\;\mathrm{terms} \end{array}$$

By inspecting Equation (2), it is observed that the polynomial that fits MSE is of the form:

(3) $$\mathrm{MSE}\left(a\right)=k_{0}\times a^{2}+k_{1}\times a+k_{2}$$

Here, the parameters $k_{0},k_{1}$ and $k_{2}$ are defined as

(4) $$\begin{array}{c} k_{0}=1(\mathrm{quadratic}\;\mathrm{term})\\ k_{1}=-2E\left[X\right](\mathrm{linear}\;\mathrm{term})\\ k_{2}=E\left[X^{2}\right](\mathrm{constant}\;\mathrm{term}) \end{array}$$

Thus, the MSE can be represented by a second-degree polynomial with quadratic coefficient 1, linear coefficient equal to −2aE[X], and constant coefficient equal to $E[X^{2}]$. □

Given that the definition of MSE involves a quadratic term of two quantities, a,b∈L, the application of a direct expansion of these terms led to the canonical form of a second-degree polynomial. This is possible because of the property of linearity of expectancies in the definition of MSE.

It is important to note that the fitting of MSE of a random variable by a second-degree polynomial is not a mere approximation. The definition of MSE is in fact in terms of a quadratic relation between elements in a random variable or collection of observations. Lemma 1 shows how to find the parameters of such a polynomial, based on the definition of MSE. Since the quadratic coefficient is always 1, we will not pay attention to it, and our focus will be on the linear and constant coefficients.

The linear coefficient of the polynomial fitting the MSE of a random variable or a distribution is equal to $k_{1}=-2E\left[X\right]=-2\mu$. We need to express $k_{2}$ in terms that we can relate both coefficients, $k_{1}$ and $k_{2}$. These two terms, $k_{1}$ and $k_{2}$, will define the foundations of a manifold that will allow comparison of distributions following a path different from that of comparing mean and standard deviations, as in state-of-the-art Information Geometry. In order to express $k_{2}$ consistently with $k_{1}$, we continue to Lemma 2.

**Lemma** **2.** *For any random variable, the constant coefficient $k_{2}$ of the polynomial fitting their MSE is equal to $\mu^{2}+\sigma^{2}$*.

**Proof.**  We have to prove that $E\left[X^{2}\right]=\mu^{2}+\sigma^{2}$, since from Lemma 1, we already know that $k_{2}=E\left[X^{2}\right]$. The variance of X is defined as

(5) $$\mathrm{Var}\left(X\right)=E\left[{\left(X-\mu \right)}^{2}\right]$$

Since μ=E[X], and $\mathrm{Var}\left(X\right)=\sigma^{2}$, and $\mathrm{Var}\left(X\right)=E\left[X^{2}\right]-{\left(E\left[X\right]\right)}^{2}$, we can rearrange it as

(6) $$\begin{array}{c} \sigma^{2}=E\left[X^{2}\right]-\mu^{2}\\ E\left[X^{2}\right]=\mu^{2}+\sigma^{2} \end{array}$$

(König–Huygens equation [14]) □

The linear and constant coefficients of the MSE-fitting polynomial ($k_{1}$ and $k_{2}$), expressed in terms of the expected value (mean) and in terms of standard deviation, are important to define our approach. These two coefficients will allow the creation of a new space in which distributions or random variables can be compared following simple differential geometry tools, as detailed in Section 3.

For simplicity, we refer to MSE(P,a) as MSE(P), again, where a refers to the observations from which P was computed. The second-degree polynomial of the MSE of a distribution P, namely MSE(P), is determined by the linear and constant coefficients, $k_{1}$ and $k_{2}$. Thus, MSE(P) refers to both the second-degree polynomial and its linear and constant coefficients (since the quadratic coefficient is always 1).

Let P and Q be two Gaussian distributions, with means $\mu_{P}$ and $\mu_{Q}$ and standard deviation $\sigma_{P}$ and $\sigma_{Q}$, respectively. Their corresponding characterization in terms of MSE are $\mathrm{MSE}\left(P\right)=[k_{1}=-2aE\left[X\right],k_{2}=\mu^{2}+\sigma^{2}]$ and $\mathrm{MSE}\left(Q\right)=[k_{1}=-2aE\left[X\right],k_{2}=\mu^{2}+\sigma^{2}]$, where X is the same range for both distributions. We observe, following Lemma 2, that MSE(P)=MSE(Q) only when their corresponding mean and standard deviations are the same.

For distributions other than Gaussians, following Lemma 1, things are not as easy. Indeed, since $k_{1}$ and $k_{2}$ are derived from the expected value of the distribution, it may occur that two distributions with the same expected value, but otherwise different, may be represented by the same parameters. This is an open problem, but for the sake of our argument, we will leave it aside for the moment and go back to it in Section 4. However, if a distribution can be approximated by its mean and standard deviations, as is the case for uniform and Poisson distributions, the approach depicted so far holds.

Our goal is to explore the properties of the space defined by the linear and constant parameters $k_{0},k_{1}$ of the second-degree polynomial fitting the MSE of probability distributions. Our approach establishes the comparison of two probability distributions P,Q not directly, for example, by computing the Aitchison or the Wasserstein distances or by computing the Fisher–Rao distance in the statistical manifold defined by the parameters of the family of distributions. Instead, we obtain for each of the compared distributions their representation as the MSE and, from it, the linear and constant coefficients of the second-degree polynomial fitting the MSE. The linear and constant coefficients $k_{0},k_{1}$ of the polynomial fitting MSE define a new structure that we will call ψ, which is a manifold in which the comparison of probability distributions can be carried out. The next section describes the properties of this manifold ψ.

The MSE(P) of a distribution P is defined by the mean and standard deviation. In that sense, the parameters $k_{0},k_{1}$, which define the linear and constant coefficients of the polynomial linked to MSE(P), can be seen as an alternative but equivalent way to define spaces or manifolds to compare distributions.

In this Section, we have revised how any probability function can be represented by its MSE. In the next section, we will review the manifold induced from the linear and constant coefficients of the second-degree polynomial describing MSE.

## 3. The Geometry of the *ψ* Manifold Induced by the Linear and Constant Parameters of MSE

The linear and constant coefficients of the second-degree polynomial describing the MSE of a distribution do not scatter randomly. In fact, as seen in Figure 2D, $k_{2}$ is nonlinearly related to $k_{1}$. The distribution of $k_{1}$ and $k_{2}$ defines a manifold that will be called ψ. In this section, some of its properties are explored. For that, we remember that $k_{1}=-2E\left[X\right]$ and $k_{2}=E\left[X^{2}\right]$. From this, it is already clear that there is a nonlinear relationship between these two coefficients. We will expand on this in the next paragraphs.

From Figure 2D and Figure 5A–E in the first column, it can be observed that the points $\left(k_{1},k_{2}\right)$ from the MSE of several probability distributions follow a clear pattern. This pattern describes a nonlinear relation between $k_{2}$ and $k_{1}$. The hypothesis is that the relationship between these two parameters can be expressed as a second degree polynomial. We want to obtain the values of α,β, and γ. For that, we present Lemma 3.

**Lemma** **3.** *The constant coefficient of the MSE-second degree fitting polynomial, $k_{2}$, can be expressed as a polynomial of second degree of $k_{1}$, that is, the linear coefficient of MSE*.

**Proof.**  To prove Lemma 3, we explicitly define the relation between $k_{2}$ and $k_{1}$:

(7) $$k_{2}=\alpha \times k_{1}^{2}+\beta \times k_{1}+\gamma$$

From Lemmas 1 and 2, we have that $k_{1}=-2\mu$, and $k_{2}=\mu^{2}+\sigma^{2}$. From here, we have that $\mu =-k_{1}/2$, and since $k_{2}=\mu^{2}+\sigma^{2}$, we can replace $k_{2}$ to reach $k_{2}={\left(\frac{-k_{1}}{2}\right)}^{2}+\sigma^{2}=\frac{k_{1}^{2}}{4}+\sigma^{2}$. From (7), it follows that $\alpha =\frac{1}{4},\beta =0,\gamma =\sigma^{2}$. □

The relation between $k_{2}$ and $k_{1}$ is defined by a polynomial of second degree, with quadratic term $\alpha =\frac{1}{4}$, linear coefficient of 0, and constant term $\gamma =\sigma^{2}$. This relation is based on the definitions of $k_{1}$ and $k_{2}$. In that sense, it is not a mere approximation but an equivalence. Equation (8) explicitly relates $k_{2}$ to $k_{1}$:

(8) $$k_{2}=\frac{1}{4}\times k_{1}^{2}+\sigma^{2}$$

We can interrogate ψ from an Information Geometry approach. The first question that arises is that of distances. Given two Gaussian distributions P and Q and represented by their corresponding parameters in the statistical manifold μ,σ, are their positions in this manifold and in ψ correlated? In other words, we want to investigate whether the Fisher–Rao distance between P and Q is somehow correlated to their distance in the assigned positions in ψ. A related question is whether the 1-D Wasserstein distance between P and P is correlated to their distance in ψ.

In order to answer these questions, we have to explicitly define a distance in the ψ space. Since our hypothesis holds, that is, there is a second-degree polynomial fitting $k_{2}$ to $k_{1}$, then a natural way to measure the distance between two points, representing two probability distributions along the curve, is to compute the arc length, which will define the geodesic between these two points.

Geodesics in ψ space are the arcs linking the points. The length of the arc from $x=x_{1}$ to $x=x_{2}$ along the curve is

$$L=\int_{x_{1}}^{x_{2}}\sqrt{1+{\left(\frac{dy}{dx}\right)}^{2}}\;dx$$

For $f\left(x\right)=\alpha \times x^{2}+\beta \times x+\gamma$:

$$f^{\prime }\left(x\right)=2\alpha x+\beta$$

It follows that

$$L=\int_{x_{1}}^{x_{2}}\sqrt{1+{\left(2\alpha x+\beta \right)}^{2}}\;dx$$

Let u=2αx+β; so, du=2αdx, and $dx=\frac{du}{2\alpha }$.

When $x=x_{1}$, $u_{1}=2\alpha x_{1}+\beta$.

When $x=x_{2}$, $u_{2}=2\alpha x_{2}+\beta$

$$L=\frac{1}{2\alpha }\int_{u_{1}}^{u_{2}}\sqrt{1+u^{2}}\;du,\;a\neq 0$$

$$\int \sqrt{1+u^{2}}\;du=\frac{1}{2}\left[u\sqrt{1+u^{2}}+\ln\left|u+\sqrt{1+u^{2}}\right|\right]+C$$

$L=\frac{1}{4\alpha }{\left[u\sqrt{1+u^{2}}+\ln\left|u+\sqrt{1+u^{2}}\right|\right]}_{u_{1}}^{u_{2}}$

We proceed to substitute u=2αx+β and we reach

(9) $$L\left(x_{1},x_{2}\right)=\frac{1}{4\alpha }{\left[\left(2\alpha x+\beta \right)\sqrt{1+{\left(2\alpha x+\beta \right)}^{2}}+\ln\left|2\alpha x+\beta +\sqrt{1+{\left(2\alpha x+\beta \right)}^{2}}\right|\right]}_{x_{1}}^{x_{2}}$$

The arc length between two points $x_{0}$ and $x_{1}$ located along a second-degree polynomial with equation $k_{2}=\alpha \times k_{1}^{2}+\beta \times k_{1}+\gamma$ is defined by Equation (9).

**Figure 3:**
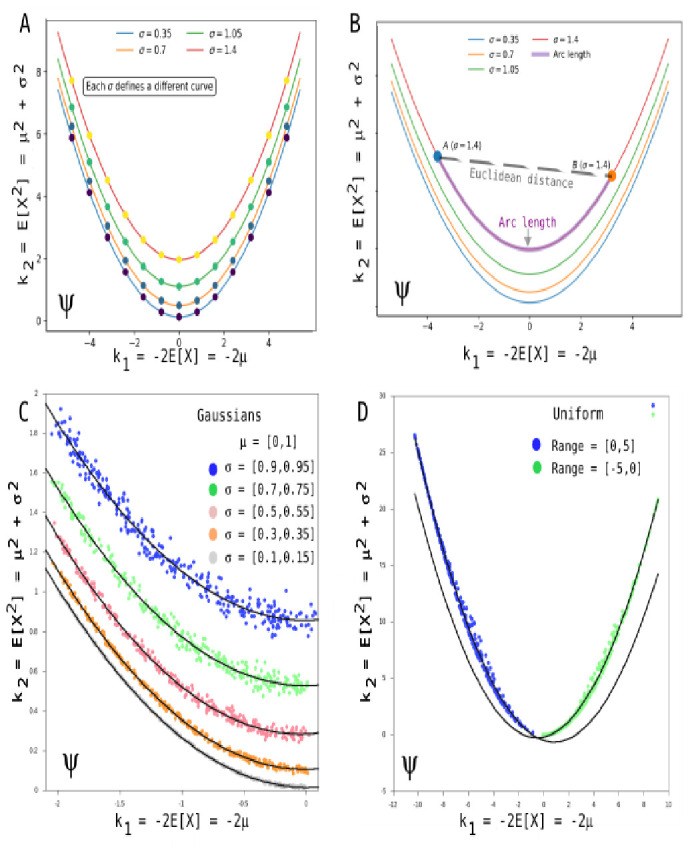
The ψ space, defined by the linear and constant coefficients of the second-degree polynomial fitting the MSE of several distributions. Each point, with coordinates $\left(k_{1},k_{2}\right)$, represents the linear ($k_{1}$) and constant ($k_{2}$) parameters that fit the MSE of a distribution. (**A**): Distributions with the same σ are shown fitting the curve in the indicated color. In (**B**), we show the geodesic for two points A and B in the ψ manifold as an arc length. For comparison, the Euclidean distance between A and B is also shown. In (**C**), representations of Gaussian distributions with mean μ∈[0,1] and σ in the specified ranges are shown. In (**D**), representations of uniform distributions in the range [−5,0] or [0,5] are shown. In all cases, the fitting polynomial for each group of distributions is shown.

The ψ manifold, defined by $k_{1}$ and $k_{2}$, is a space that allows the comparison of distributions. In order to reveal more of its structure, Figure 3 shows several examples of distributions represented by their corresponding coefficients $k_{1}$ and $k_{2}$ as the coordinates in ψ. In Figure 3A, the different curves represent the second degree polynomial for distributions with the same σ. Distributions in the same curve and thus with equal σ may have a different μ, which is coded in the coordinate $k_{1}$. In Figure 3B, we show the geodesic between points A and B, which corresponds to the arc length of the polynomial shown in Equation (8). For contrast, the Euclidean distance between A and B is also shown.

Figure 3C, shows several distributions, obtained from Gaussians with μ∈[0,1] and σ in the indicated ranges. In contrast with Figure 3A, here, the distributions, shown as colored points, present small variations in their σ. Each point, representing a distribution, has as its coordinates $k_{1}$ and $k_{2}$, where, as discussed in the previous Section, a probability distribution P is represented by $\mathrm{MSE}\left(P,a\right)=a^{2}+k_{1}\times a+k_{2}$. Distributions with similar σ fit the same curve. Each point represents a distribution of 1000 samples. In Figure 3D, the case for uniform distributions is shown, for samples drawn from the specified intervals. Again, each point represents a distribution consisting of 1000 observations.

The curvature κ of a structure is of high relevance in Differential Geometry. The curvature of a function f at point x is defined as [15,16]

(10) $$\kappa \left(x\right)=\frac{|f^{\prime \prime }\left(x\right)|}{{\left[1+{\left(f^{\prime }\left(x\right)\right)}^{2}\right]}^{3/2}}$$

The curvature of a polynomial of second degree such as those defined by Equation (8), is defined as

(11) $$\kappa \left(k_{1}\right)=\frac{1}{2{\left(1+\frac{k_{1}^{2}}{4}\right)}^{3/2}}$$

**Figure 4:**
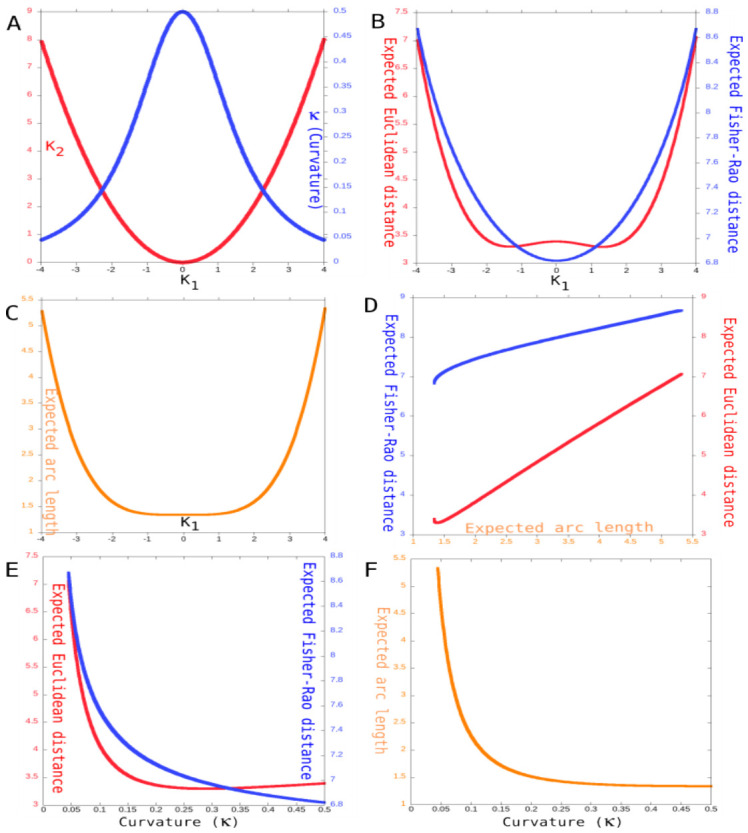
The curvature of ψ and the relation between distances and between curvature and distances. (**A**): The ψ manifold (red) and the curvature of each point on it (blue). (**B**): For each point in a discrete version of ψ, the expected Euclidean distance to the rest of points in ψ was computed and shown in red, as a function of $k_{1}$. In blue, it is shown the expected Fisher-Rao distance (see Equation (12)) between the μ and σ coordinates of each point and the rest, in the (μ,σ) manifold. (**C**): The expected arc length as a function of $k_{1}$ is displayed. (**D**): The relation between Euclidean distance (in the ψ manifold) and the arc length in psi is shown in red. In blue, we show the relation between the Fisher–Rao distance (in the (μ,σ) manifold) and the arc length in ψ. (**E**): The expected Euclidean (in the ψ manifold) and Fisher–Rao (in the (μ,σ) manifold) distances as a function of the curvature of ψ. (**F**): The expected arc length of a point in ψ as a function of the curvature of that point in ψ.

The curvature κ of ψ is defined in terms of $k_{1}$ and $k_{2}$, the linear and constant coefficients of the second degree polynomial fitting the MSE of a distribution. A natural question to ask is whether the notion of distance or geodesics is affected by the curvature. First, Figure 4 shows ψ (red) and the curvature associated with each point along it (blue), as defined in Equation (11). As is clear from the definition of a curvature over a parabola, the maximum curvature corresponds to the point of the vanishing derivative.

Once the curvature of ψ is explicitly presented (Figure 4A), we can proceed in the path to offer an answer to the question of how geodesics in ψ are affected by curvature. In Figure 4B, we show the expected Euclidean distance (red) from each point in ψ (only the $k_{1}$ coordinate is displayed) to the rest of points in ψ. Along it, in blue, we show the expected Fisher–Rao distance for the associated point in the (μ,σ) manifold. Each point in ψ can be easily mapped to the (μ,σ) manifold since $k_{1}$ and $k_{2}$ are defined in terms of μ and σ (see Equations (2) and (7)). The expected Fisher–Rao distance has its minimum at the lowest point of the parabola, whereas the minimum expected Euclidean distance of points in ψ does not correspond to the lowest point of ψ. This comes from the definition of Euclidean distance and will not be discussed here. For both the Euclidean and Fisher–Rao distances, it is observed that points closer to the vertex of ψ tend to present a lower expectancy, with the exception of the already mentioned increase in the expected Euclidean distance for the vertex in ψ.

Figure 4C presents the expected arc length in ψ as a function of $k_{1}$. Here, as for the Euclidean and Fisher–Rao distances, it is observed that points located in the extremes of ψ present a larger expected arc length. Interestingly, close to the vertex, the expected ar length decrease slower than for the Fisher–Rao and Euclidean distances. This suggests that the arc length is less affected by the curvature. In order to give more evidence of such assertion, Figure 4D shows the relation between the expected Euclidean (red) and Fisher-Rao (blue) distances with the expected arc length for points in ψ. For the Euclidean case, the relation is almost linear, except for points in ψ that are indeed close to the vertex ($k_{1}∼0$), where the expected Euclidean distance increases. The Fisher–Rao relation with the arc length is increasing, although nonlinear.

The effect of the curvature in the distances is shown in Figure 4E,F. In Figure 4E, the expected Euclidean (red) and Fisher–Rao (blue) distances are shown as a function of the curvature κ of ψ. It is observed that the expected Fisher–Rao distance is monotonically decreasing with the curvature of ψ. On the other hand, the expected Euclidean distance presents a different behavior, as it decreases and then increases as a function of the curvature. Finally, in Figure 4F, the expected arc length is presented as a function of the curvature κ(ψ). The expected arc length of points in ψ is decreasing with the curvature, although, as observed, the effect of curvature is asymptotic. This means that the arc length of points in ψ can be used to compare distributions even when the curvature of the associated parameters is relatively large. So far, this is only a conjecture, but if it holds, then our proposal would be useful to compare distributions whose coordinates in ψ do present a large curvature.

A comparison of the distributions in ψ is straightforward, as long as the distributions are located along the curve. From Equation (8) and Figure 3A, it is observed that distributions with the same σ will be located in the same ψ structure. If this is the case, the arc length that separates the points in the curve offers a natural distance function. If distributions do not have the same σ, our approach offers no better solutions to the existing ones from Information Geometry. We will expand into these limitations and other properties of the approach defined here in Section 4.

In this section, we revised the space defined by the linear and constant coefficients of the second-degree polynomial of the mean square error of a probability distribution. We observed that the distribution of these two parameters for several probability distributions is not random and, in some cases, can be fitted by a second-degree polynomial.

## 4. Computational Explorations

Most of the applications of Information Geometry deal with large collections of data. There, the parameters have to be inferred from data, and theoretical results, although valuable, are not directly applied. For instance, first and second moments of the distributions are to be computed from data. In that sense, it is relevant to explore the performance of our proposed with existing theoretical tools, such as the Fisher–Rao distance.

The Fisher–Rao distance between two Gaussian distributions P and Q, with mean $\mu_{P}$, $\mu_{Q}$ and standard deviation $\sigma_{P}$ and $\sigma_{Q}$, respectively, defined in the statistical manifold induced by μ,σ, is given by [8,17,18]

(12) $$FR\left(P,Q\right)=2\times \sqrt{2}arctanh\sqrt{\frac{{\left(\mu_{P}-\mu_{Q}\right)}^{2}+2\times {\left(\sigma_{P}-\sigma_{Q}\right)}^{2}}{{\left(\mu_{P}-\mu_{Q}\right)}^{2}+2\times {\left(\sigma_{P}+\sigma_{Q}\right)}^{2}}}$$

Since the goal of this contribution is to verify whether the ψ space offers an interesting perspective at the comparison of probability distributions, a natural question to ask is whether the Fisher–Rao distance in the parameter space (μ,σ) manifold is correlated to the arc length in ψ. A second relevant question is whether the 1-D Wasserstein distance between the histograms is correlated with the arc length (geodesic) in the ψ manifold.

**Figure 5:**
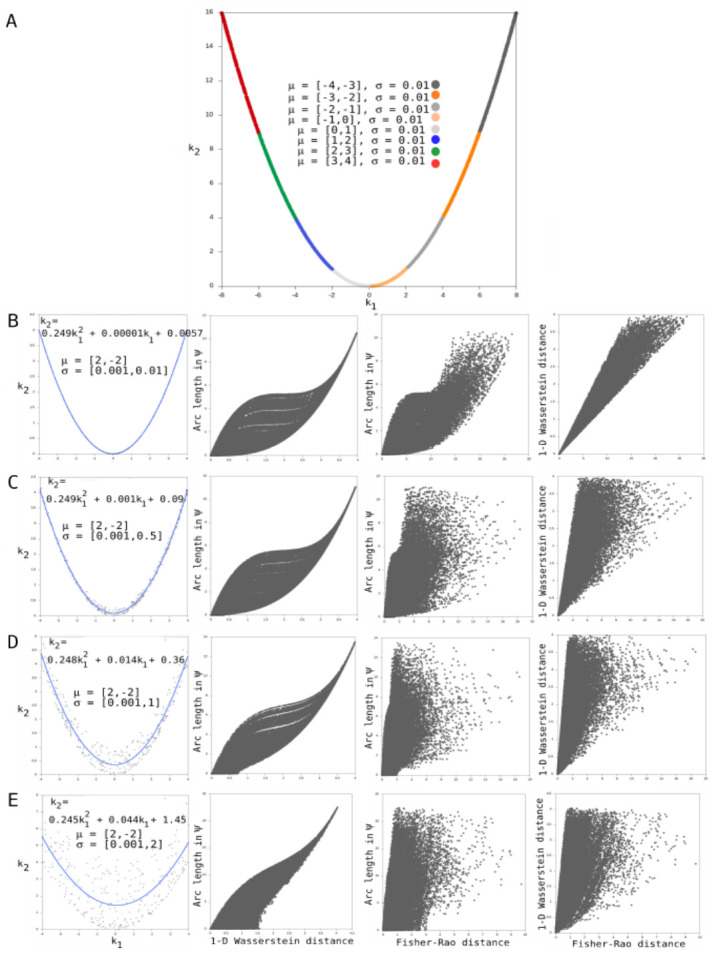
The ψ manifold. This space is generated by the linear ($k_{1}$) and constant ($k_{2}$) coefficients of the MSE-fitting polynomial. (**A**): The distribution of Gaussian distributions with mean μ in the range [−2,2] and the indicated σ in the ψ space: for each of the ranges for μ, 200 distributions were obtained, consisting of 1000 measurements or observations. From (**B**–**E**): Each gray dot represents a Gaussian distribution with increasing range of σ. The first column shows ψ and the fitting polynomial in blue (See Equation (7)). The second column shows the the arc length joining the corresponding points of distributions in ψ as a function of the the Wasserstein distance of of the corresponding histograms. The third column shows also the arc length as a function of the Fisher–Rao distance in the μ,σ manifold. The last columns show how the 1-D Wasserstein distance varies with the Fisher–Rao distance.

To gather empirical evidence to answer the two previous questions, we conducted several experiments. Figure 5 shows several Gaussian distributions and their characterization. In Figure 5A, we show the coordinates $k_{0},k_{1}$ of hundreds of Gaussian distributions with mean in the specified range and a very low σ=0.001. The points, that is, the distributions represented by $k_{0},k_{1}$ closely fit the theoretical polynomial $k_{2}=\frac{1}{4}\times k_{1}^{2}+0\times k_{1}+\sigma^{2}$.

In Figure 5B–E, the ψ space (first column) is shown again for Gaussian distributions with a mean in the range [−2,2] and increasing range for σ. The distributions or, more specifically, the parameters $k_{1}$ and $k_{2}$ that define the MSE of the distributions are shown as gray dots. Each dot is a Gaussian with mean in the range [−2,2] and standard deviation in the indicated range. The solid blue line is the second-degree polynomial that fits $k_{1}$ and $k_{2}$. From Equation (7) and the subsequent text, we have $k_{2}=\alpha \times k_{1}^{2}+\beta \times k_{1}+\gamma$, and $\alpha =\frac{1}{4},\beta =0,\gamma =\sigma^{2}$.

For the first case shown in Figure 5B, first column, and from the generated data when σ∈[0.001,0.01], we have $k_{2}=0.249\times k_{1}^{2}+0.000001\times k_{1}+0.0057$. When the range of variation for σ increases ([0.001,0.5]), as shown in Figure 5C, the equation of the polynomial fitting the points is $k_{2}=0.249\times k_{1}^{2}+0.001\times k_{1}+0.09$. When σ varies in a wider range ([0.001,1]), the equation is $k_{2}=0.248\times k_{1}^{2}+0.014\times k_{1}+0.36$ (Figure 5D). Finally, when σ is allowed to vary even more ([0.001,2]), the polynomial is $k_{2}=0.245\times k_{1}^{2}+0.044\times k_{1}+1.45$ (Figure 5E).

The parameter γ increases with σ, as expected from Equation (7). It is also observed that, as σ increases, the points are more scattered along the fitting polynomial, which is expected because distributions can present a wider variance. Finally, even though β was supposed to be null, from the derivation of Equation (7), when working with samples, it presents a value greater than 0 and increasing with σ.

In the second column of Figure 5B–E, the arc length, computed by Equation (9), is shown as a function of the 1-D Wasserstein distance. It is clear that the correlation is not perfect, although a peculiar pattern appears. The relation between the Fisher–Rao distance (FR, Equation (12)) in the statistical manifold induced by μ,σ and the arc length, shown in the third column, is far from perfect. This speaks of the different nature of comparing probability distributions in the two manifolds. The last column shows the scatter plot of 1-D Wasserstein distance and the Fisher–Rao distance. It is observed that for distributions with a wider range for σ, the correlation between Wasserstein and FR decreases.

In this section, we offered computational evidence that the comparison in the ψ space, that is, the space induced by the linear and constant parameters of the second degree polynomial of MSE, may offer interesting alternatives to compare distributions.

## 5. Discussion and Conclusions

The comparison of probability distributions is of high relevance in many fields. The comparison can be directly applied over the distributions as with the Aitchison, Wasserstein, or other relevant distances. Alternatively, a comparison can be conducted in terms of the parameters that describe the distributions. Those parameters may define a statistical manifold, as is the case for Gaussian distributions when their mean and standard deviations are considered. There, the Fisher–Rao distance can be applied, since it captures the inherent geometry of the data points. There are other possibilities that attempt a parametrization of probability distributions, with a different list of relevant parameters.

In this contribution, we have described how a discrete probability function P can be described by its mean square error, or MSE, along its range. MSE(P) can be approximated by a second-degree polynomial $k_{0}\times a^{2}+k_{1}\times a+k_{2}$, where $k_{0}=1$, $k_{2}=-2aE\left[X\right]$, and $k_{3}=E\left[X^{2}\right]$, where a∈L, and where *L* is the set of observations or measurements from which P is computed.

The linear and constant parameters of the MSE-fitting polynomial define an interesting structure. The distribution of $k_{1}$ and $k_{2}$ defines a nonlinear pattern. In particular, $k_{2}$ can be approximated by a second-degree polynomial from $k_{1}$ with parameters $\frac{1}{4},0,\sigma^{2}$ for the quadratic, linear and constant terms for certain cases, for example, for distributions with similar parameters.

Every probability distribution is mapped to a point along the second-degree polynomial that relates the linear and constant parameter of the MSE-fitting polynomial. The comparison between probability distributions in this space can be conducted by computing the arc length of the curve defined by the polynomial. We presented empirical evidence of the possible relevance of such a comparison. Counting with a clear picture of how probability distributions are related to each other and thus how to best compare them is still an open question. Our aim is to offer more details on this comparison by the proposed method.

The approach introduced in this contribution is closely related to the existing methods in Information Geometry. There, distributions are mapped to a space defined by first and second statistical momenta, namely the mean and standard deviation. In that space, distributions can be compared by an appropriate function, such as the Fisher–Rao distance. Our approach is similar to other methods in the sense that it works with the same statistical momenta. However, our approach diverges from others in the sense that distributions are transformed to the mean square error representation. The mean square error representation of a distribution is a second degree polynomial, relating the mean and the standard deviation of the distribution. The linear and constant parameters of this representation define a manifold in which distributions can be compared by computing the arc length joining them.

Our approach, as shown by the numerical and analytical evidence, does not arrive at the same results as the existing schemes. The manifold introduced in this contribution, where distributions can be compared, is different to the usual manifold considered in Information Geometry, although the parameters are the same. In that sense, it is an alternative that may be of interest in cases in which distributions can be approximated by first and second statistical momenta.

Comparing probability functions that cannot be adequately represented by μ and σ is still an open problem [9]. In this contribution, we explored alternative paths that allow a comparison between probability distributions of different families, while focusing on the mean and standard deviation. We do so by obtaining the mean square error of the studied probability functions, and then, we fit a second-degree polynomial to the mean square error of the probability functions. The parameters of such a polynomial define a manifold, which can also be approximated by a second degree polynomial. The comparison of probability functions can then be conducted in the latter polynomial. The position of every probability function is a point along the polynomial-defined manifold; so, the length of the arc that separates the points that represent the probability distributions is a natural way to compare such probability functions.

There are several possible extensions to the ideas presented here. More theoretical results are in order, for example, consider third or higher momentum in the error measure, as opposed to what is present in the mean square error, which takes into account only first and second momenta.

An analytical derivation of the expected arc length as a function of the curvature in the ψ manifold is a natural next step. Counting with such a function and comparing this to the corresponding one for the Euclidean and Fisher–Rao distance would make a fairer comparison between the approach introduced here and the existing ones. The empirical and numerical evidence we have presented opens the door for more research.

## Data Availability

The software associated with this contribution is available at https://anomalocarisproject.github.io, accessed on 27 April 2026. No data other than synthetic were used for this publication.
